# Transforming and facilitating health care delivery through social networking platforms: evidences and implications from WeChat

**DOI:** 10.1093/jamiaopen/ooae047

**Published:** 2024-05-30

**Authors:** Jiancheng Ye

**Affiliations:** Weill Cornell Medicine, New York, NY, United States

**Keywords:** telehealth, global health, health care delivery, patient-generated health data, social networking platform

## Abstract

**Objectives:**

Telehealth or remote care has been widely leveraged to provide health care support and has achieved tremendous developments and positive results, including in low- and middle-income countries (LMICs). Social networking platform, as an easy-to-use tool, has provided users with simplified means to collect data outside of the traditional clinical environment. WeChat, one of the most popular social networking platforms in many countries, has been leveraged to conduct telehealth and hosted a vast amount of patient-generated health data (PGHD), including text, voices, images, and videos. Its characteristics of convenience, promptness, and cross-platform support enrich and simplify health care delivery and communication, addressing some weaknesses of traditional clinical care during the pandemic. This study aims to systematically summarize how WeChat platform has been leveraged to facilitate health care delivery and how it improves the access to health care.

**Materials and Methods:**

Utilizing Levesque’s health care accessibility model, the study explores WeChat’s impact across 5 domains: Approachability, Acceptability, Availability and accommodation, Affordability, and Appropriateness.

**Results:**

The findings highlight WeChat’s diverse functionalities, ranging from telehealth consultations and remote patient monitoring to seamless PGHD exchange. WeChat’s integration with health tracking apps, support for telehealth consultations, and survey capabilities contribute significantly to disease management during the pandemic.

**Discussion and Conclusion:**

The practices and implications from WeChat may provide experiences to utilize social networking platforms to facilitate health care delivery. The utilization of WeChat PGHD opens avenues for shared decision-making, prompting the need for further research to establish reporting guidelines and policies addressing privacy and ethical concerns associated with social networking platforms in health research.

## Introduction

The COVID-19 pandemic prompted many countries to intensify their focus on enhancing health care systems. However, for low- and middle-income countries (LMICs), providing modern and cost-effective services, such as sophisticated health care delivery platforms common in high-income countries (HICs), posed a considerable challenge during the global public health emergency. In response, the adoption of telehealth or remote care gained widespread acceptance, aiming to improve the accessibility of health care services in LMICs and resource-limited settings.^1–3^

A novel health communication paradigm, social media or social networking platforms, emerged, enabling individuals to communicate, share information about their health and health conditions, and exchange health-related messages. The proliferation of smartphones, including in LMICs, coincided with the rapid growth of social media.^4^ Utilizing social networking platforms for health communication not only facilitates the availability and sharing of health information but also proves convenient for delivering peer, social, and emotional support.^5^ Recognizing the potential, public health agencies acknowledged the utility of social media platforms to engage patients and enhance the reach and efficiency of public health services, including surveillance, research, and communication.^6^^,^^7^ For instance, Facebook served as a tool to promote public health and implement educational health services.^8^ Similarly, Twitter was also utilized to advance psychological and mental health support.^9–11^

**Figure 1.**
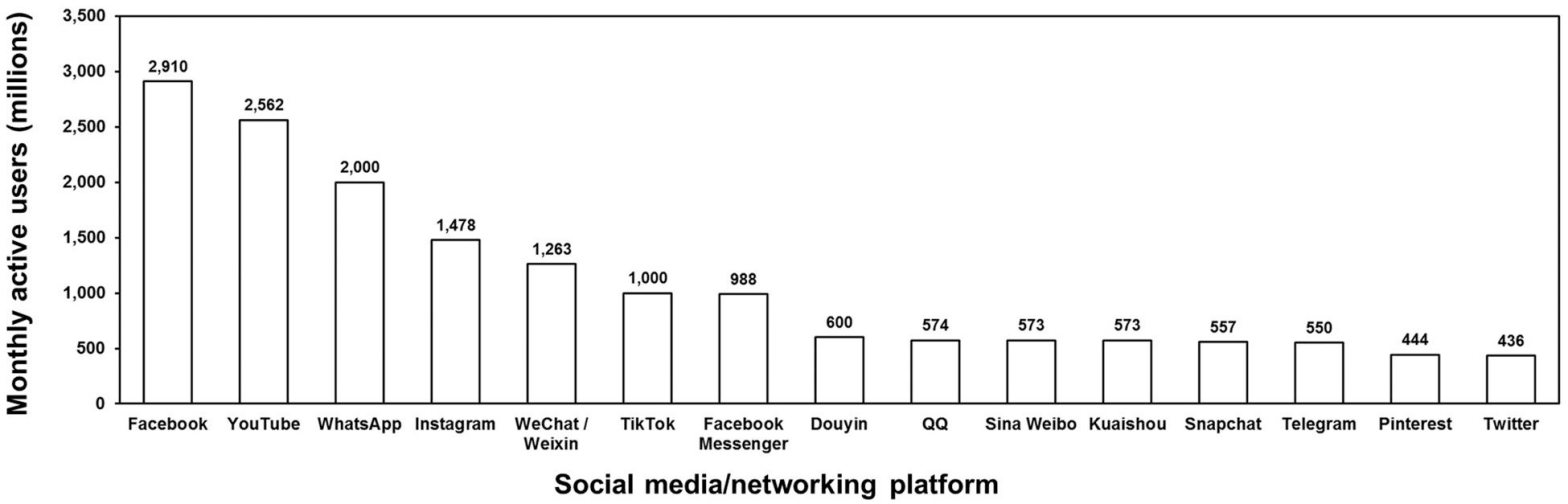
Global social media/networking platform active users 2022 (Top 15).

WeChat (or “Weixin” in China) is one of the most popular social networking platforms. Initially launched in 2011 as a mobile messaging app by the Chinese company Tencent, WeChat has evolved into one of the leading social networks globally.^12^Figure 1 illustrates the top 15 global social media/networking platforms’ active users ranking,^13^ with WeChat securing the fifth position. The only platforms ranking higher are 3 Facebook-related applications/platforms (Facebook, WhatsApp, and Instagram) and YouTube. Notably, other well-known platforms like Twitter and LinkedIn fall below WeChat in terms of active users. WeChat’s widespread acceptance can be attributed to its numerous interactive functions. Presently, the number of WeChat users exceeds 900 million, with 150 million users spending at least 2 hours online daily.^14^ Additionally, more than 200 million users have linked WeChat accounts with credit cards, 70% of whom spend more than $15 per month on services provided by WeChat.^14^

### WeChat and patient-generated health data

Patient-generated health data (PGHD) refers to health-related information that is collected, recorded, and shared by individuals.^15^ PGHD encompasses data that patients generate outside of traditional health care settings, such as hospitals or clinics.^16^ PGHD can be gathered through various devices, applications, or platforms, including wearable devices, mobile health apps, patient portals, or self-reported surveys. It is gaining broader adoption across the health care spectrum, holding significant promise for improving care and health outcomes.^17^^,^^18^

Simultaneously, the rapid evolution of social media has created an environment where the creation and transmission of personal health information are easy, quick, and convenient for patients. Social media and networking platforms offer numerous opportunities for patients, their families, and friends to share personal health-related information.^19^^,^^20^

**Figure 2.**
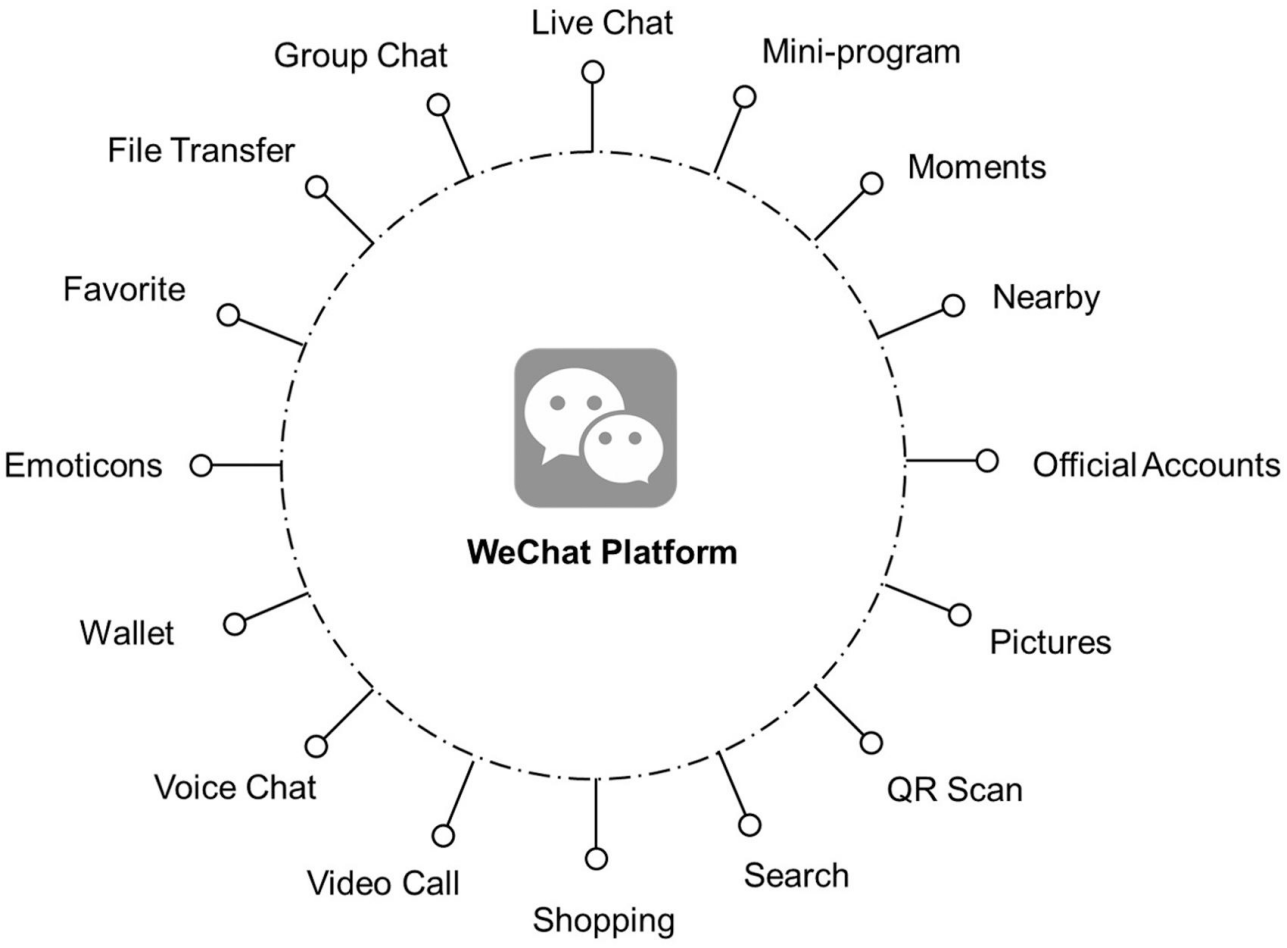
Health-related functions on WeChat platform.

WeChat, with its diverse functions and services, serves as a platform that also collects rich PGHD. Users can send instant messages in various formats such as text, images, videos, and audio to friends, family, and groups. Figure 2 illustrates the health-related functions provided by the WeChat platform. Many users access WeChat “Moments” regularly, enabling them to share short videos, post status updates, including emojis and stickers, similar to Facebook’s status and newsfeed. It facilitates comments on shared posts, including the “like” function. WeChat also offers social networking functions like online shopping, games, and city services. Voice and text messaging, group messaging, payment, and games are additional popular WeChat functions. WeChat official accounts, applied by individuals or official institutions (eg, centers for disease control and prevention, community health care centers, hospitals) on the WeChat Public Platform, provide a platform for cooperation, promotion services, open and equal access, enhanced interaction, data statistics, automatic responses, development platforms, and other features.^21^ The following function allows users to follow favorite accounts. During the COVID-19 pandemic, many Chinese people used WeChat to submit health reports and scan health monitoring quick-response (QR) codes to enter or leave public places.^22^^,^^23^

We classified the functions into 5 domains based on the health-related services that they provide:

- **Education and knowledge dissemination:** Favorite, File Transfer, Group Chat, Live Chat, Mini-program, Official Accounts, Search, Video Call, Voice Chat.
- **Social networking and mental health support**: Emoticons, Group Chat, Live Chat, Mini-program, Moments, Nearby, Pictures, QR scan, Search, Shopping, Video Call, Voice Chat, Wallet.
- **Consultation:** Group Chat, Live Chat, Mini-program, Video Call, Voice Chat.
- **Monitoring and triage:** Mini-program, Pictures, QR Scan, Video Call, Voice Chat.
- **Clinical care and follow-up:** Group Chat, Live Chat, Mini-program, Official Accounts, Video Call, Voice Chat.

Table 1 illustrates the definitions of the Taxonomy in the WeChat-driven health care delivery system. WeChat, not only an innovative mobile application ingrained in most individuals’ daily routines, serves as an indispensable tool transforming users’ lives in various aspects. Amid the COVID-19 pandemic, the remote management of diseases necessitates the virtual exchange of health-related data between patients and clinicians.^24^ WeChat has become a hub for a diverse range of PGHD that users create, record, gather, and transfer. For instance, patients can send PGHD, such as blood pressure or blood glucose readings, to clinicians through WeChat private or group chats using text or photos. WeChat also seamlessly integrates with third-party health tracking apps, enabling users to connect their health monitoring devices and share collected data, including steps taken, blood pressure, heart rate, or sleep patterns, with health care providers or store it as part of the patient’s health record.^25^

**Table 1.** WeChat-driven health care delivery system taxonomy and definitions.

Term/concepts	Definition
WeChat	A social networking platform that integrates various services such as messaging, socialization, mobile payment services, and consistently expands its functionality by incorporating new services, including health services.
WeChat service	The integration of offline and online services into the mobile terminal to provide location tracking, appointment pre-check, business processing, online payment, progress inquiry, evaluation, complaints, etc.
Service retention	The proportion of users who use the WeChat service again within a certain time duration.
Quality of service	Characteristics of the service that meet specified and potential needs, ensuring it aligns with the predetermined service level to satisfy the recipients’ demands.
Star rating system for service quality	WeChat service’s rating system used to determine whether the service can be launched, optimized, or upgraded to an excellent benchmark service. Domains include the degree of adaptation, compatibility, integration, and support of related systems.
Health care service users	WeChat users who utilize health-related services provided by the WeChat platform, which may or may not be directly connected with traditional clinical settings.
WeChat health users	WeChat users who use health-related services provided by WeChat.
Electronic health insurance card	The electronic version of the traditional health insurance card. It is embedded in the user’s WeChat account, and users can make payments anytime and anywhere.
Electronic health treatment card	The electronic version of the traditional health treatment card. It is embedded in the user’s WeChat account, and users can use it to register, make appointments, make payments, order and check medication, and share information with health care workers.
Card-bound user	Users who bind the health insurance card in WeChat and use the health insurance card through WeChat.

Furthermore, health care organizations or researchers can leverage WeChat’s built-in survey and questionnaire functions to collect patient-reported data. Patients can respond to health-related surveys, self-assessments, or outcome measures, providing valuable insights into their health status or treatment outcomes. WeChat serves as a platform for telehealth consultations, allowing patients to have virtual appointments with health care providers. During these consultations, patients can share information about their symptoms, provide updates on their condition, or even share images or videos related to their health concern, thereby generating relevant PGHD.^26^ These characteristics position WeChat as an effective telehealth or remote care platform, particularly during the pandemic. Many Chinese users have linked their health insurance accounts with WeChat, facilitating direct payments through the platform. WeChat’s user-friendly service evaluation services enable patients to identify high-quality health care services.^27^

Additionally, WeChat functions as a vital platform to verify authorized information sources.^28^ For instance, the verification process for official accounts allows organizations, public figures, and businesses to authenticate their accounts and display a verification badge. This badge assists users in identifying official accounts of trustworthy entities, including health care organizations, government agencies, or reputable news outlets. Users can subscribe to specific official accounts that offer health information or health care services, ensuring access to reliable content and updates. WeChat’s search feature, called WeChat Index, enables users to search for articles, official accounts, and other content within the platform. This feature aids users in finding information from verified accounts and gauging the popularity and engagement of content. Users can leave comments, ratings, and feedback on articles and official account posts, allowing them to assess the credibility and quality of information provided by different sources.^29^

### WeChat and access to health care

Access to health care is a fundamental aspect of the performance of health care systems globally, particularly in resource-limited settings such as LMICs.^30^ The COVID-19 pandemic has exacerbated challenges for patients in maintaining access to care.^31^ We mapped the services that WeChat provides on Levesque’s model, which was designed to explain the comprehensiveness and dynamic nature of access to health care with 5 domains of accessibility (Approachability, Acceptability, Availability and accommodation, Affordability, and Appropriateness).^32^Table 2 outlines the domains and definitions of WeChat-driven health care services, accompanied by an illustration of the specific services within each domain.

**Table 2.** WeChat-driven health care services domains.

Domains	Definition	WeChat-driven health care service
Approachability	People facing health needs can identify that some form of services exists, can be reached, and has an impact on the individual’s health.	WeChat serves as an effective tool to disseminate knowledge, change patients’ unhealthy habits, and provide regular health education.
Acceptability	Cultural and social factors determining the possibility for people to accept the aspects of the service and the judged appropriateness for individuals to seek care.	Clinicians can update electronic health information based on patient feedback and adjust treatment plans according to patients’ health condition. Clinicians can strengthen relationships with patients through WeChat, engaging in shared decision-making to foster better outcomes.
Availability and accommodation	Health services (physical space or health care roles) can be reached both physically and in a timely manner.	Many health care services can be accessed through WeChat, improving medical service quality, reducing emissions from traffic, consuming fewer resources, and being more cost-effective.
Affordability	Economic capacity for people to spend resources and time to use appropriate services.	WeChat application can be downloaded and used free of charge. Hospitals or community health centers can create separate WeChat groups or official accounts for different diseases, providing real-time and continuous health services.
Appropriateness	The fit between services and clients’ needs, timeliness, the amount of care spent in assessing health problems, determining correct treatment, and the technical and interpersonal quality of services provided.	The WeChat-driven health care delivery model can enhance the clinician-patient relationship by providing individualized health instructions and improving dynamic communications. Shared decision-making allows effective experiences and measures to be widely learned.

#### Approachability

Patients can access medical knowledge, health information, and official educational materials through WeChat’s education modules.^33^ WeChat facilitates direct consultation with health care providers, enabling patients to obtain professional support and address issues promptly without the need for in-person visits, alleviating the inconvenience of travel for office visits.

#### Acceptability

WeChat serves as an empowering platform, facilitating collaborative health management between patients, caregivers, and clinicians through shared decision-making that considers patients’ preferences. The integration of group communication functions improves communication efficiency, enabling clinicians to gain a deeper understanding of a patient’s health status over time. This, in turn, results in a reduction in office visits and hospital readmissions, ultimately enhancing patient outcomes and experiences.^34^

Health care providers can update electronic health information based on patient feedback, adapting treatment plans in real-time. This dynamic approach mobilizes patient enthusiasm, transforming passive static management into an active, responsive model. WeChat provides an accessible medium for patients to inquire, seek clarifications, or discuss concerns, thereby enhancing the acceptability of health care services through direct and convenient communication. The platform’s appointment booking features simplify the scheduling process, reduce wait times, and enhance overall patient experience. WeChat’s official accounts feature empowers health care organizations to share reliable health information, educational content, and updates with their followers. This accessibility promotes health literacy, empowers patients, and fosters a better understanding of health conditions and treatment options, thereby improving the overall acceptability of health care services. WeChat facilitates the creation of patient support groups and communities, connecting individuals with similar health conditions or concerns. These communities offer emotional support,^35^ encourage self-management, and enhance the acceptability of health care by fostering a sense of belonging and understanding. Users can leave feedback, comments, and ratings on health care services, official accounts, or articles, providing a platform for patients to express opinions, share experiences, and contribute to the continual improvement of the quality and acceptability of health care services.^36^

#### Availability and accommodation

WeChat facilitates the creation of disease-specific groups, enabling patients and health care providers to deliver real-time and continuous health services. This approach allows health care providers to guide patients anytime and anywhere. WeChat’s diverse forms of information exchange and popular social networking features break the traditional text exchange model, increasing patient engagement with minimal technology literacy challenges.^37^^,^^38^

Moreover, the platform serves as a versatile tool for requesting prescription refills and accessing medical records. Patients can communicate with health care providers through the app, facilitating requests for necessary medications and providing real-time access to their health history. This function, akin to a patient portal, serves as a comprehensive platform for health information, appointment scheduling, prescription refills, and consultation services.^39^

#### Affordability

The cost of WeChat is low because the WeChat application can be downloaded and used free of charge. WeChat is available in most countries in the world and provides more than 17 languages.^14^ Its user-friendly interface ensures usability even for the elderly population, allowing direct message sending and reading without technological challenges.^40^ WeChat integrates seamlessly with health insurance platforms, providing users with access to information about coverage, claims, or policy details. This integration aids individuals in understanding their insurance benefits, comparing costs, and making informed decisions about health care services in consideration of their affordability. In China, certain telehealth services covered by national insurance allow users to make payments directly through WeChat Pay.^41^

WeChat contributes to proactive health care management by providing valuable information on preventive measures, healthy lifestyles, and disease management. Its official accounts feature empowers health care organizations and professionals to share educational content. Users leverage the platform to share health-related information, articles, and tips.^28^ Official health organizations or experts can create accounts, offering accurate and reliable health information to encourage users to adopt healthier habits.^19^ Users can create personal health tracking within WeChat, logging daily activities, exercise routines, diet, and mood.^42^ This function enables users to track progress over time, make adjustments based on their goals, and receive reminders for medication, exercise sessions, and regular health check-ups through WeChat’s reminder and notification features. WeChat further supports the creation of fitness challenge groups, allowing users to invite friends to participate in various fitness activities and competitions.^43^

The platform integrates with various third-party health management apps, empowering users to track health metrics, monitor chronic conditions, and manage medications. This proactive approach in health management may potentially prevent complications and reduce health care costs associated with acute episodes or exacerbations. Health care providers and organizations can leverage WeChat to promote affordable health care services, discounts, or special offers, enabling users to discover cost-effective options for treatments, screenings, or wellness services, thereby enhancing health care affordability and accessibility.^44^

#### Appropriateness

The WeChat-based follow-up model plays a pivotal role in cultivating a stronger clinician-patient relationship by providing individualized health instructions and enhancing dynamic communications.^37^^,^^45^ This approach facilitates the identification of patient care concerns and enables targeted interventions. Furthermore, WeChat efficiently gathers patient information, creating an extensive database that offers valuable data support for enhancing diagnosis and treatment procedures. Through this method, WeChat enables continuous, timely, and comprehensive follow-up interventions, ultimately contributing to a reduction in morbidity, mortality, and disability rates associated with chronic diseases.^46^ This functionality aligns with the overarching goal of promoting proactive health care.^47^ Patients or their families can choose appropriate communication methods through WeChat based on their needs. Non-urgent matters can be addressed by leaving messages for health care providers via text, while urgent concerns can be addressed directly with health care providers through voice or video calls. WeChat’s official accounts feature empowers trustworthy health care organizations and professionals to share accurate and evidence-based health information. By accessing reliable information through WeChat, users can make informed decisions about their health, understand appropriate treatment options, and learn about preventive measures. WeChat excels in facilitating care coordination and referrals between health care providers.^48^ It enables efficient communication and information sharing, ensuring appropriate transitions of care and guaranteeing that patients receive the necessary and appropriate services across various health care settings.

WeChat enhances patient engagement by providing a platform for individuals to actively participate in their health care decisions. Secure messaging allows patients to communicate their preferences, concerns, and treatment goals, promoting shared decision-making and ensuring that health care aligns with their needs and values. WeChat integrates seamlessly with health monitoring devices or apps, allowing patients to collect and share their own health data. This PGHD offers valuable insights to health care providers, enabling more personalized and appropriate care decisions based on the patient's individual health information.

### Integration of WeChat data and electronic health record

#### Integration process

**Figure 3.**
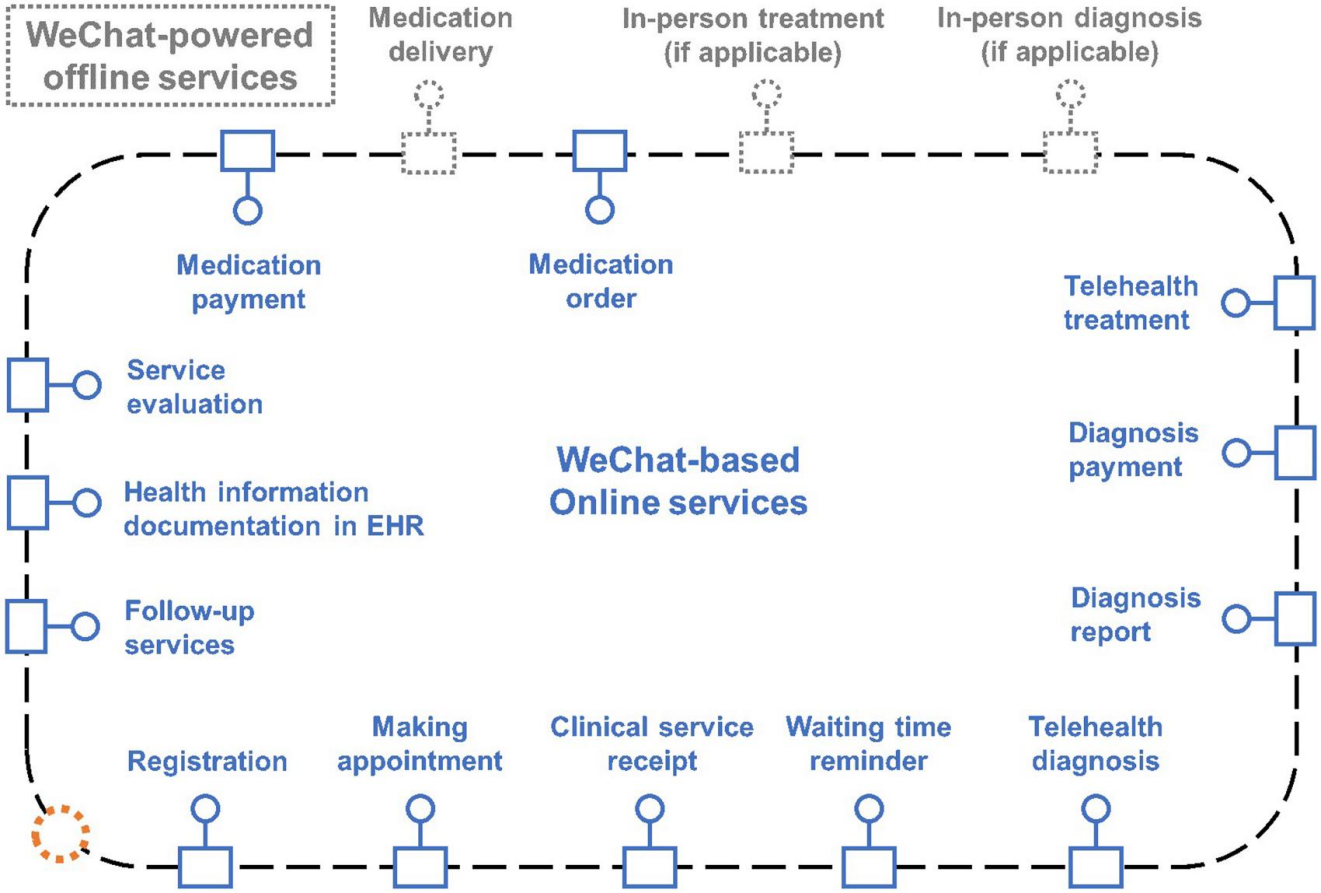
WeChat-driven health care delivery system workflow.

WeChat plays a crucial role in effectively collecting patient information, forming a comprehensive database that provides valuable data support for optimizing diagnosis and treatment. The workflow of the WeChat-driven health care delivery system is illustrated in Figure 3. Integrating PGHD from WeChat with electronic health records (EHRs) in hospitals yields numerous benefits. The inclusion of patient-reported outcomes and real-time information on the platform enriches data, complementing clinical decision support based on EHRs. Stakeholders can develop mini-programs, electronic questionnaires, video health lectures, and public health accounts to generate and collect health data.

Establishing a standardized health information exchange (HIE) and management system, characterized by simplicity and automatic data capture, necessitates collaboration among policymakers, health institutions, and the WeChat platform. Key efforts include the implementation of identifiers to amalgamate WeChat PGHD and EHRs, integration of terminology and messaging standards, formulation of policies and regulations governing the integration mechanism, and development of strategies to address data privacy.

WeChat serves as a medium for sending medication reminders and notifications to patients. Integration with an EHR system enables the synchronization of relevant medication information, including dosage, frequency, and special instructions, with the patient’s EHR.^49^ This integration improves medication adherence and provides a clearer understanding of the patient’s medication history.^50^ Noteworthy practices during the pandemic in China have demonstrated successful integration of WeChat PGHD and EHR data.^22^^,^^47^^,^^51^ WeChat’s capabilities extend to scheduling and managing health care appointments. Integration with an EHR system facilitates the seamless transfer of appointment details, such as date, time, and location, from WeChat to the patient’s EHR. This integration streamlines administrative processes and consolidates relevant information in one centralized location. WeChat’s secure messaging and video call platform can be integrated with an EHR system, enabling health care providers to securely communicate with patients, share test results, discuss treatment plans, and conduct virtual consultations. The conversation history and pertinent details are logged within the patient’s EHR, ensuring continuity of care.

#### Security and privacy

The integration process may encounter obstacles due to legal and regulatory concerns related to data ownership and usage. Safeguarding the security and privacy of WeChat PGHD poses challenges for patients, clinicians, and researchers. WeChat PGHD is susceptible to security breaches, compromising data integrity and exposing it to potential misuse, as it currently operates outside established security regulatory frameworks.^52^ Concerns include insecure points of data collection and insecure data movement, which may expose the device or users’ WeChat accounts to potential risks such as unauthorized access and cyber threats.

WeChat, lacking end-to-end encryption, raises security considerations. While it does offer some encryption for messages, its current encryption primarily secures messages during transmission between the user’s device and the server, as well as between the servers themselves. Unlike end-to-end encryption, where only the communicating parties can decrypt messages, WeChat has the technical capability to access and decrypt user messages on their servers. While messages are encrypted during transmission, the service provider could potentially access them. Establishing standards for data representation and transmission is crucial for creating a secure environment for WeChat-based health services, paralleling developments in other health information technologies. Transparency in WeChat’s data ownership and use policies is vital for informed decision-making by patients and providers regarding the creation and transmission of PGHD. WeChat developers must enhance transparency to enable discussions about patient expectations and provider concerns, fostering mutual benefits from the use of WeChat PGHD.

In instances where patients shared health information, they had the option to choose between group chat or private chat with health care providers, and personal health advice was sent via private chat. The health information recorded by pre-assigned health care providers into the EHR was accessible only to researchers for further analysis.^53^^,^^54^ Patients will be was informed about the study process in advance. To address potential security concerns like lost phones, patients could log into WeChat from another device and promptly change their password, ensuring the security of personal information. However, there are still existing limitations in the current privacy and security management system, such as ensuring the prevention of third-party use of patient numbers for phishing or other attacks^55^

Considering the rapid increase in the use of social networking platforms in health care, especially during the pandemic, China has taken steps to protect personal information.^56^ The adoption of the Personal Information Protection Law (PIPL) in China, effective November 1, 2021,^57^ is similar to the Health Insurance Portability and Accountability Act (HIPAA) in the United States. The PIPL aims to safeguard personal information rights, standardize personal information handling activities, and promote the rational use of personal information.^58^

### Limitations

While WeChat offers certain advantages for health care delivery, it also has limitations. First, WeChat predominantly functions as a messaging and social media platform, making it less conducive to complex health consultations. Although it facilitates basic communication between patients and health care providers, it may lack the features necessary for comprehensive health evaluations, diagnoses, or treatment planning. Second, WeChat lacks a robust system for verifying the credentials of health care providers on the platform. This absence of verification raises concerns about the qualifications and expertise of individuals offering medical advice or services through WeChat. Patients may find it challenging to differentiate between legitimate health care professionals and unqualified individuals, potentially compromising the quality and safety of care. Third, while WeChat is widely used in China, its reach and accessibility may be limited outside of the country. Health care regulations and licensing requirements vary between countries and regions. WeChat’s health care delivery capabilities may be subject to regulatory restrictions or limitations in certain jurisdictions, hindering its widespread adoption for health consultations or telemedicine services. Compliance with local laws and regulations is crucial for ensuring patient safety and maintaining the standard of care. International patients or those residing in areas where WeChat is not prevalent may face difficulties accessing health care services or receiving timely responses from health care providers using the platform. Forth, WeChat has faced criticism for its privacy and security practices, including concerns about data collection and government surveillance. When it comes to sensitive health information, such as medical records or personal health data, maintaining privacy and security is of utmost importance. The potential risks associated with data breaches or unauthorized access to health information may deter individuals from fully trusting WeChat as a platform for health care delivery. Health care organizations and relevant stakeholders should assess the platform’s capabilities and compliance with local regulations to determine whether it meets the specific requirements of their health care delivery context.

## Conclusion

Social networking platforms have become a valuable resource for health care delivery, especially for LMICs. WeChat spearheads a new era of mobile communication in health care delivery. Importantly, a variety of health-related information is continuously generated and transmitted among large numbers of users through WeChat’s different functions, enabling WeChat enormous potential to affect the general public’s health status. The use of WeChat PGHD facilitates the shared decision-making between patients and clinicians, along with patients’ understanding of and adherence to treatment plans. Research on how social media or social networking platforms transform the public’s health status is highly necessary. Future endeavors should focus on developing standardized reporting guidelines for health research conducted on social networking platforms, including WeChat. Additionally, the establishment of policies addressing privacy and ethical concerns is imperative for the responsible and ethical use of these platforms in health care research and delivery.

## Data Availability

The data underlying this article are available in the article.

## References

[1] Walker PGT , Whittaker C, Watson OJ, et al The impact of COVID-19 and strategies for mitigation and suppression in low-and middle-income countries. Science. 2020;369(6502):413-422. 32532802 10.1126/science.abc0035 PMC7292504

[2] Hoffer-Hawlik MA , Moran AE, Burka D, et al Leveraging telemedicine for chronic disease management in low-and middle-income countries during COVID-19. Glob Heart. 2020;15(1):63. 33150128 10.5334/gh.852 PMC7500231

[3] Ye J. The role of health technology and informatics in a global public health emergency: practices and implications from the COVID-19 pandemic. JMIR Med Inform. 2020;8(7):e19866. 32568725 10.2196/19866 PMC7388036

[4] Silver L , Huang C, Taylor K. In Emerging Economies, Smartphone and Social Media Users Have Broader Social Networks. Pew Research Center; 2019.

[5] Moorhead SA , Hazlett DE, Harrison L, et al A new dimension of health care: systematic review of the uses, benefits, and limitations of social media for health communication. J Med Internet Res. 2013;15(4):e1933. 10.2196/jmir.1933 PMC3636326 23615206

[6] Thackeray R , Neiger BL, Smith AK, et al Adoption and use of social media among public health departments. BMC Public Health. 2012;12(1):242-246. 22449137 10.1186/1471-2458-12-242 PMC3331826

[7] Ye J , Hai J, Wang Z, et al Leveraging natural language processing and geospatial time series model to analyze COVID-19 vaccination sentiment dynamics on tweets. JAMIA Open. 2023;6(2):ooad023. 37063408 10.1093/jamiaopen/ooad023 PMC10097455

[8] Kite J , Foley BC, Grunseit AC, et al Please like me: Facebook and public health communication. PLoS One. 2016;11(9):e0162765. 27632172 10.1371/journal.pone.0162765 PMC5025158

[9] Gabarron E , Wynn R. Use of social media for sexual health promotion: a scoping review. Glob Health Action. 2016;9(1):32193. 27649758 10.3402/gha.v9.32193 PMC5030258

[10] Coppersmith G , Dredze M, Harman C. Quantifying mental health signals in Twitter. In: *Proceedings of the Workshop on Computational Linguistics and Clinical Psychology: From Linguistic Signal to Clinical Reality*, Baltimore, MD; June 27, 2014:51-60.

[11] Ye J. Pediatric mental and behavioral health in the period of quarantine and social distancing with COVID-19. JMIR Pediatr Parent. 2020;3(2):e19867. 32634105 10.2196/19867 PMC7389340

[12] Statistics: WeChat monthly active users (MAU) 2011-2022. https://www.chinainternetwatch.com/31608/wechat-statistics/

[13] We Are Social, H., & DataReportal. (January 26, 2022). Most popular social networks worldwide. Digital 2022: Global digital overview. https://wearesocial.com/cn/wp-content/uploads/sites/8/2022/01/DataReportal-GDR002-20220126-Digital-2022-Global-Overview-Report-Essentials-v02.pdf

[14] WeChat Wiki. https://wechatwiki.com/

[15] Deering MJ , Siminerio E, Weinstein S. *Issue Brief: Patient-Generated Health Data and Health IT*. Office of the National Coordinator for Health Information Technology; 2013:20.

[16] Tiase VL , Hull W, McFarland MM, et al Patient-generated health data and electronic health record integration: a scoping review. JAMIA Open. 2020;3(4):619-627. 33758798 10.1093/jamiaopen/ooaa052 PMC7969964

[17] Shapiro M , Johnston D, Wald J, Mon D. Patient-generated health data. *RTI International*. 2012;813:814.

[18] Ye J. The impact of electronic health record–integrated patient-generated health data on clinician burnout. J Am Med Inf Assoc. 2021;28(5):1051-1056. 10.1093/jamia/ocab017 PMC8068436 33822095

[19] Ye J , Wang Z, Hai J. Social networking service, patient-generated health data, and population health informatics: national cross-sectional study of patterns and implications of leveraging digital technologies to support mental health and well-being. J Med Internet Res. 2022;24(4):e30898. 35486428 10.2196/30898 PMC9107051

[20] Sinnenberg L , Buttenheim AM, Padrez K, et al Twitter as a tool for health research: a systematic review. Am J Public Health. 2017;107(1):e1-e8. 10.2105/AJPH.2016.303512 PMC5308155 27854532

[21] Zhang Y , Xia T, Huang L, et al Factors influencing user engagement of health information disseminated by Chinese provincial centers for disease control and prevention on WeChat: observational study. JMIR Mhealth Uhealth. 2019;7(6):e12245. 31250833 10.2196/12245 PMC6620885

[22] Xu H , Huang S, Qiu C, et al Monitoring and management of home-quarantined patients with COVID-19 using a WeChat-based telemedicine system: retrospective cohort study. J Med Internet Res. 2020;22(7):e19514. 32568727 10.2196/19514 PMC7333794

[23] Zhang Q-L , Xie W-P, Lei Y-Q, et al Telemedicine usage via WeChat for children with congenital heart disease preoperatively during COVID-19 pandemic: a retrospective analysis. Int J Qual Health Care. 2021;33(2):mzab066. 33835158 10.1093/intqhc/mzab066 PMC8083340

[24] Ye J , He L, Beestrum M. Implications for implementation and adoption of telehealth in developing countries: a systematic review of China’s practices and experiences. NPJ Digit Med. 2023;6(1):174. 37723237 10.1038/s41746-023-00908-6 PMC10507083

[25] Ye J , Li N, Lu Y, et al A portable urine analyzer based on colorimetric detection. Anal Methods. 2017;9(16):2464-2471.

[26] Ye J. Patient safety of perioperative medication through the lens of digital health and artificial intelligence. JMIR Perioper Med. 2023;6:e34453. 37256663 10.2196/34453 PMC10267793

[27] Sun M , Yang L, Chen W, et al Current status of official WeChat accounts for public health education. J Public Health (Oxf). 2021;43(3):618-624. 31974552 10.1093/pubmed/fdz163

[28] Zhang X , Wen D, Liang J, et al How the public uses social media WeChat to obtain health information in China: a survey study. BMC Med Inform Decis Mak. 2017;17(Suppl 2):66-79. 28699549 10.1186/s12911-017-0470-0 PMC5506568

[29] Ye J , Woods D, Bannon J, et al Identifying contextual factors and strategies for practice facilitation in primary care quality improvement using an informatics-driven model: framework development and mixed methods case study. JMIR Hum Factors. 2022;9(2):e32174. 35749211 10.2196/32174 PMC9269526

[30] Ye J , Xiong S, Wang T, et al The roles of electronic health records for clinical trials in low-and middle-income countries: scoping review. JMIR Med Inform. 2023;11:e47052. 37991820 10.2196/47052 PMC10701650

[31] Ye J , Orji IA, Baldridge AS, et al; Hypertension Treatment in Nigeria Program Investigators. Characteristics and patterns of retention in hypertension care in primary care settings from the hypertension treatment in Nigeria program. JAMA Netw Open. 2022;5(9):e2230025. 36066896 10.1001/jamanetworkopen.2022.30025 PMC9449788

[32] Levesque J-F , Harris MF, Russell G. Patient-centred access to health care: conceptualising access at the interface of health systems and populations. Int J Equity Health. 2013;12(1):18-19. 23496984 10.1186/1475-9276-12-18 PMC3610159

[33] Zeng F , Deng G, Wang Z, et al WeChat: a new clinical teaching tool for problem-based learning. Int J Med Educ. 2016;7:119-121. 27111920 10.5116/ijme.5708.e5c4 PMC4844534

[34] Liu J , Zheng X, Chai S, et al Effects of using WeChat-assisted perioperative care instructions for parents of pediatric patients undergoing day surgery for herniorrhaphy. Patient Educ Couns. 2018;101(8):1433-1438. 29499997 10.1016/j.pec.2018.02.010

[35] Ye J. Advancing mental health and psychological support for health care workers using digital technologies and platforms. JMIR Form Res. 2021;5(6):e22075. 34106874 10.2196/22075 PMC8274671

[36] Ye J. Design and development of an informatics-driven implementation research framework for primary care studies. In: *AMIA Annual Symposium Proceedings*. American Medical Informatics Association; 2021. PMC8861697 35308925

[37] Lyu K-X , Zhao J, Wang B, et al Smartphone application WeChat for clinical follow-up of discharged patients with head and neck tumors: a randomized controlled trial. Chin Med J (Engl). 2016;129(23):2816-2823. 27900995 10.4103/0366-6999.194635 PMC5146789

[38] Ye J , et al 2023. Multimodal data hybrid fusion and natural language processing for clinical prediction models. medRxiv, p. 2023.08. 24.23294597. 10.1101/2023.08.24.23294597 PMC11141806 38827058

[39] Sakumoto M , Ye J, Kalu R, et al Patient portal perceptions in an urban community health center setting: Insights for telehealth. THMT. 2022;7(5):373.

[40] Li X , Li T, Chen J, et al A WeChat-based self-management intervention for community middle-aged and elderly adults with hypertension in Guangzhou, China: a cluster-randomized controlled trial. Int J Environ Res Public Health. 2019;16(21):4058. 31652688 10.3390/ijerph16214058 PMC6862068

[41] Chen N. Stakeholder power analysis of the facilitators and barriers for telehealth solution implementation in China: a qualitative study of individual users in Beijing and interviews with institutional stakeholders. JMIR Form Res. 2022;6(1):e19448. 35044321 10.2196/19448 PMC8811689

[42] Ye J , Ma Q. The effects and patterns among mobile health, social determinants, and physical activity: a nationally representative cross-sectional study. In: *AMIA Annual Symposium Proceedings*. American Medical Informatics Association; 2021. PMC8378627 34457181

[43] Li Y , Zhou Y, Chen M, et al A WeChat-based rehabilitation platform for children and adolescents with congenital heart disease to promote cardiac fitness (heartfit): protocol for a mixed-methods strategy from evidence-based design to pilot study. J Multidiscip Healthc. 2022;15:907-920. 35519154 10.2147/JMDH.S349519 PMC9064066

[44] Ye J , Ren Z. Examining the impact of sex differences and the COVID-19 pandemic on health and health care: findings from a national cross-sectional study. JAMIA Open. 2022;5(3):ooac076. 36177395 10.1093/jamiaopen/ooac076 PMC9494404

[45] Ye J , Sanuade OA, Hirschhorn LR, et al Interventions and contextual factors to improve retention in care for patients with hypertension in primary care: hermeneutic systematic review. Prev Med. 2024;180:107880. 38301908 10.1016/j.ypmed.2024.107880 PMC10919242

[46] Ye J , et al 2024. Development and application of natural language processing on unstructured data in hypertension: a scoping review. medRxiv, p. 2024.02. 27.24303468. 10.1101/2024.02.27.24303468

[47] Chen X , Zhou X, Li H, et al The value of WeChat application in chronic diseases management in China. Comput Methods Programs Biomed. 2020;196:105710. 32858284 10.1016/j.cmpb.2020.105710

[48] Ye J , Zhang R, Bannon JE, et al Identifying practice facilitation delays and barriers in primary care quality improvement. J Am Board Fam Med. 2020;33(5):655-664. 32989060 10.3122/jabfm.2020.05.200058

[49] Feinglass J , Wang JA, Ye J, et al Hospital care for opioid use in Illinois, 2016–2019. J Behav Health Serv Res. 2021;48(4):597-609. 33502670 10.1007/s11414-020-09748-8 PMC7839292

[50] Baldridge AS , Aluka-Omitiran K, Orji IA, et al Hypertension treatment in Nigeria (HTN) program: rationale and design for a type 2 hybrid, effectiveness, and implementation interrupted time series trial. Implement Sci Commun. 2022;3(1):84-18. 35918703 10.1186/s43058-022-00328-9 PMC9344662

[51] Liu G , Wang S, Liao J, et al The efficacy of WeChat-based parenting training on the psychological well-being of mothers with children with autism during the COVID-19 pandemic: quasi-experimental study. JMIR Ment Health. 2021;8(2):e23917. 33481751 10.2196/23917 PMC7879717

[52] Wager KA , Lee FW, Glaser JP. Health Care Information Systems: A Practical Approach for Health Care Management. John Wiley & Sons; 2021.

[53] Ye J , Sanchez-Pinto LN. Three data-driven phenotypes of multiple organ dysfunction syndrome preserved from early childhood to middle adulthood. In: *AMIA Annual Symposium Proceedings*. American Medical Informatics Association; 2020. PMC8075454 33936511

[54] Ye J , Yao L, Shen J, et al Predicting mortality in critically ill patients with diabetes using machine learning and clinical notes. BMC Med Inform Decis Mak. 2020;20(Suppl 11):295-297. 33380338 10.1186/s12911-020-01318-4 PMC7772896

[55] Ienca M , Vayena E. On the responsible use of digital data to tackle the COVID-19 pandemic. Nat Med. 2020;26(4):463-464. 32284619 10.1038/s41591-020-0832-5 PMC7100462

[56] Ye J. Health information system’s responses to COVID-19 pandemic in China: a national cross-sectional study. Appl Clin Inform. 2021;12(2):399-406. 34010976 10.1055/s-0041-1728770 PMC8133837

[57] Personal Information Protection Law of the People’s Republic of China. https://en.wikipedia.org/wiki/Personal_Information_Protection_Law_of_the_People%27s_Republic_of_China

[58] Act A. Health insurance portability and accountability act of 1996. Public Law. 1996;104:191. 16477734

